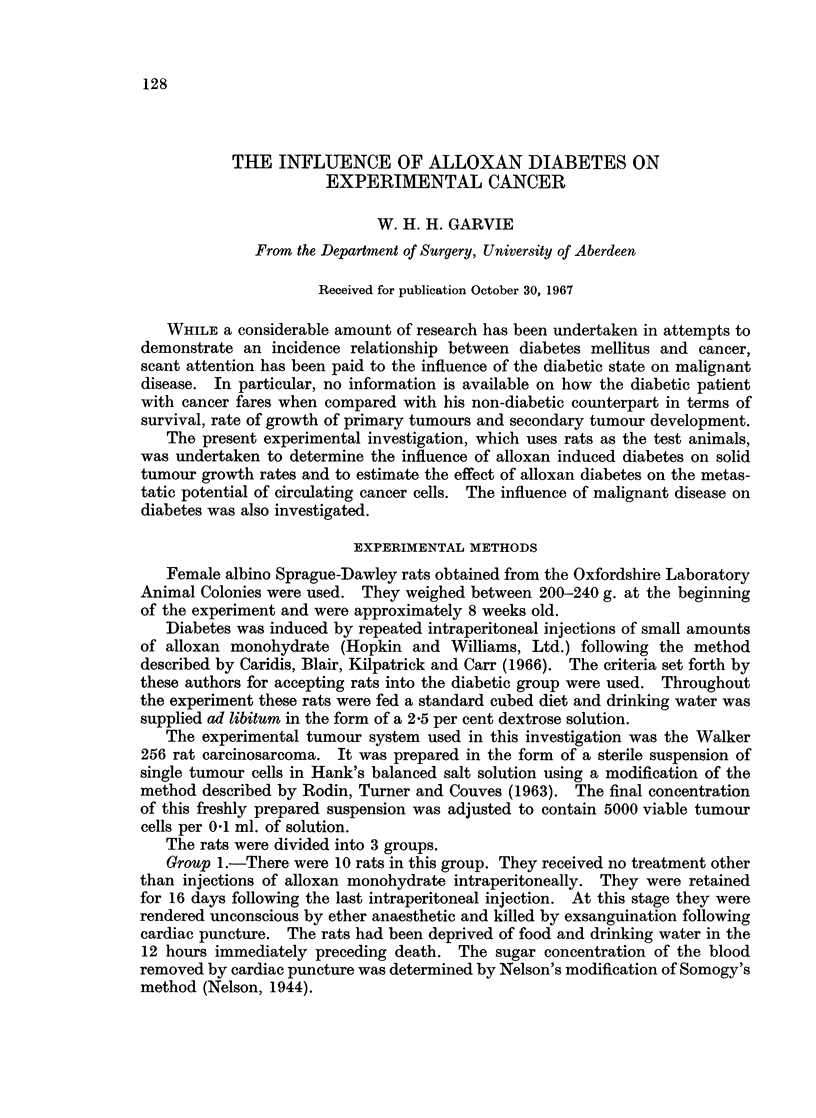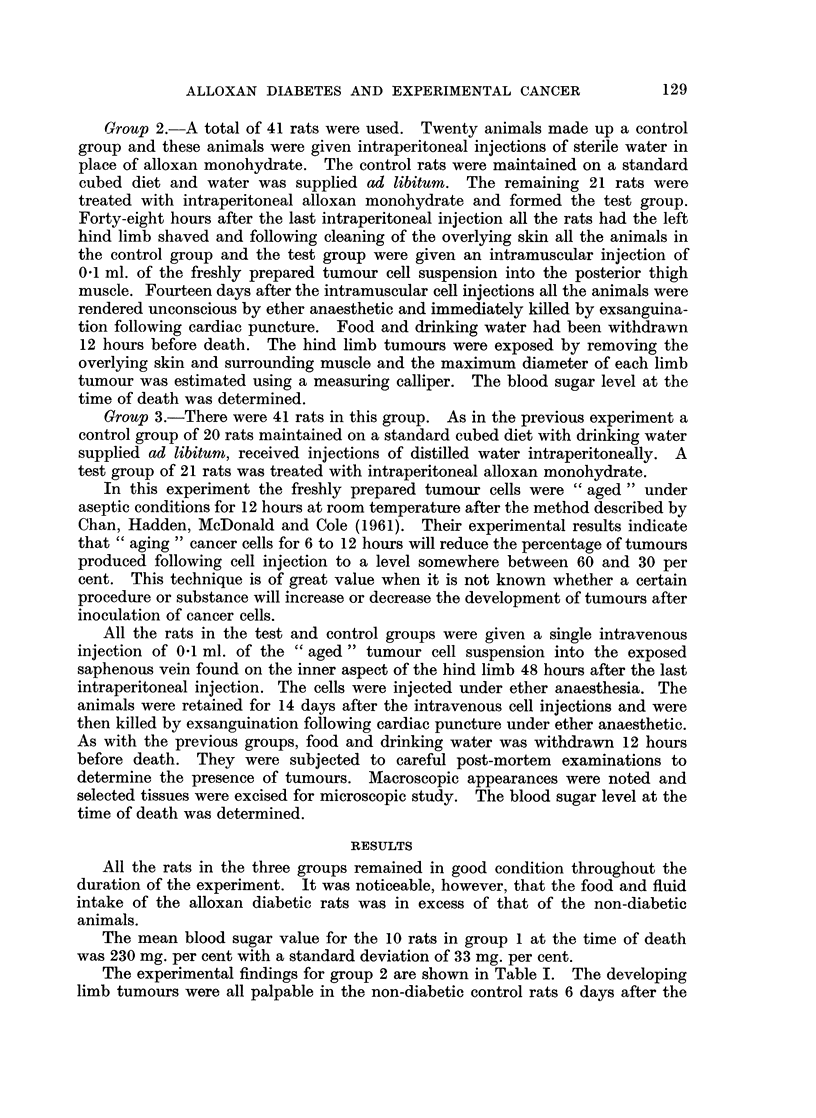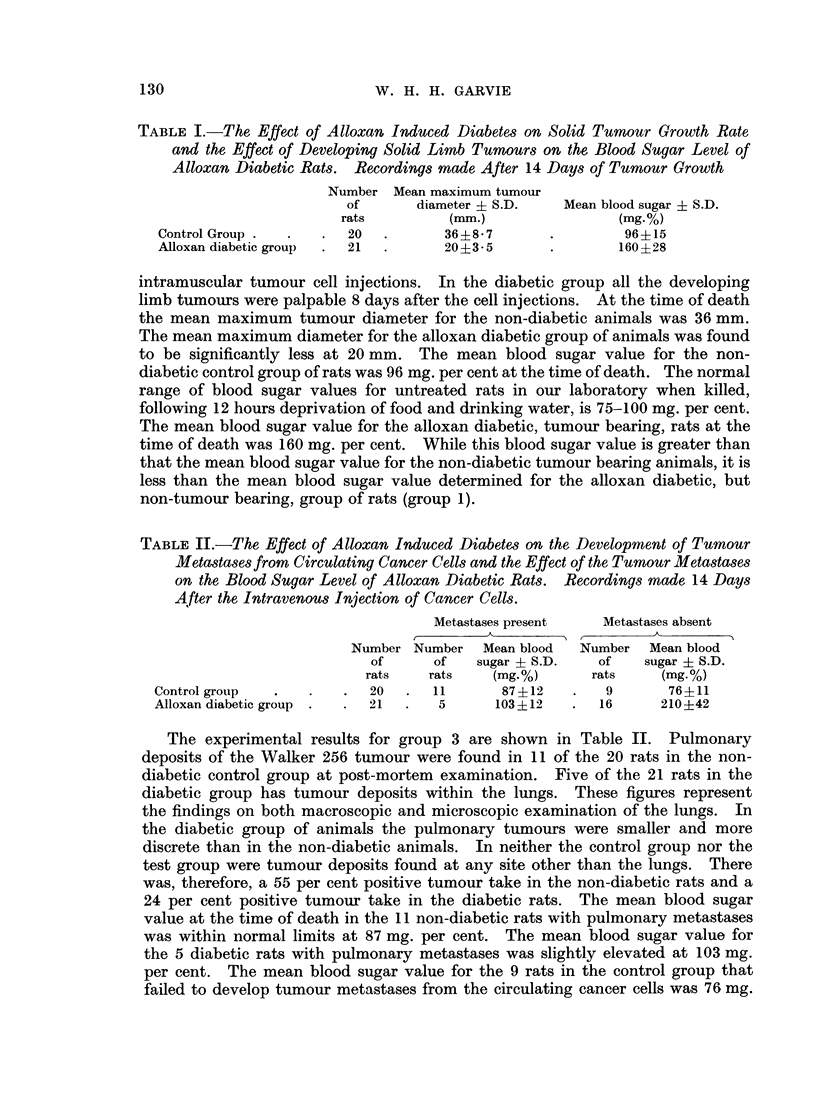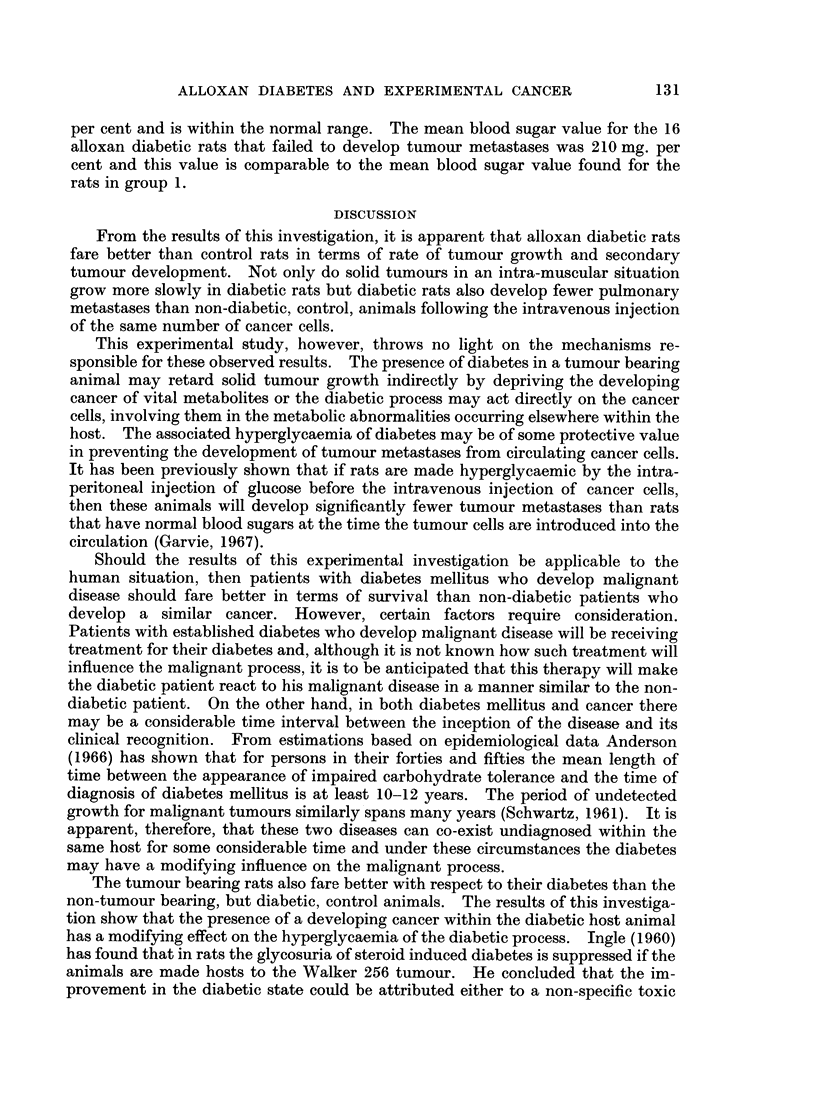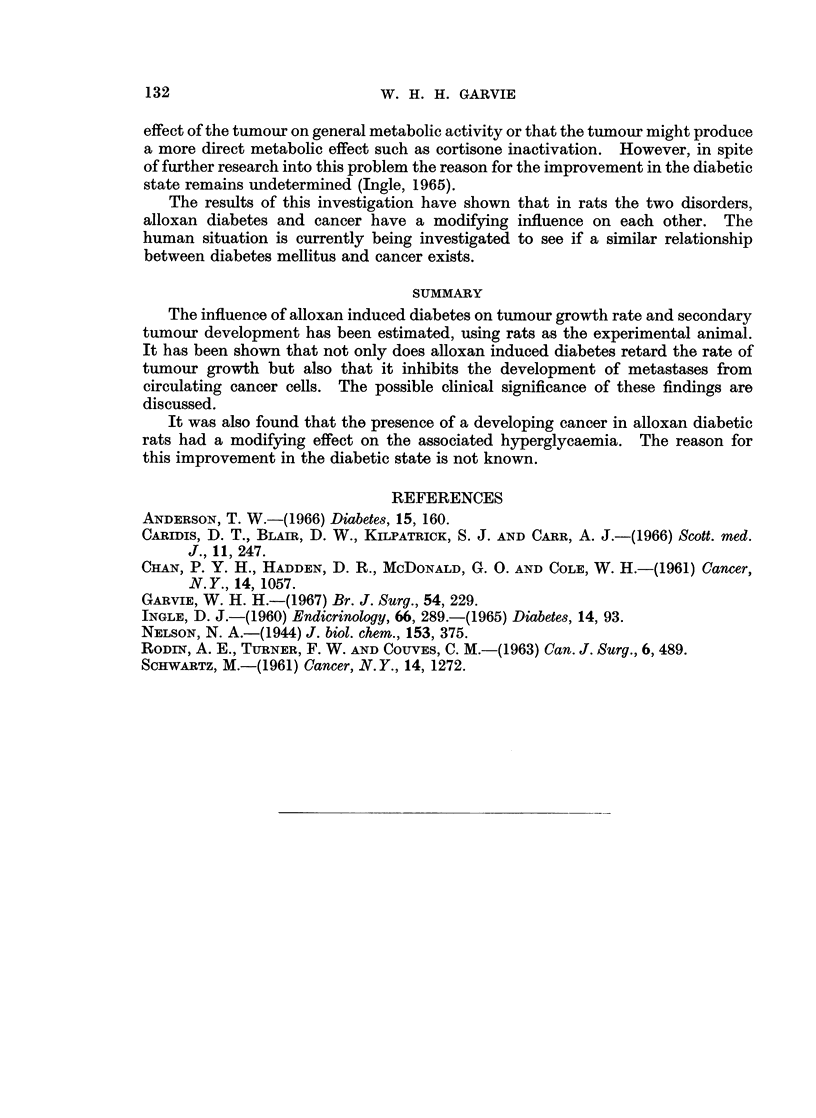# The influence of alloxan diabetes on experimental cancer.

**DOI:** 10.1038/bjc.1968.18

**Published:** 1968-03

**Authors:** W. H. Garvie


### THE INFLUENCE OF ALLOXAN DIABETES ON EXPERIMENTAL CANCER

W. H. H. GARVIE

*From the Department of Surgery, University of Aberdeen*

Received for publication October 30, 1967

WHILE a considerable amount of research has been undertaken in attempts to demonstrate an incidence relationship between diabetes mellitus and cancer, scant attention has been paid to the influence of the diabetic state on malignant disease. In particular, no information is available on how the diabetic patient with cancer fares when compared with his non-diabetic counterpart in terms of survival, rate of growth of primary tumours and secondary tumour development.

The present experimental investigation, which uses rats as the test animals, was undertaken to determine the influence of alloxan induced diabetes on solid tumour growth rates and to estimate the effect of alloxan diabetes on the metastatic potential of circulating cancer cells. The influence of malignant disease on diabetes was also investigated.

### EXPERIMENTAL METHODS

Female albino Sprague-Dawley rats obtained from the Oxfordshire Laboratory Animal Colonies were used. They weighed between 200–240 g. at the beginning of the experiment and were approximately 8 weeks old.

Diabetes was induced by repeated intraperitoneal injections of small amounts of alloxan monohydrate (Hopkin and Williams, Ltd.) following the method described by Caridis, Blair, Kilpatrick and Carr (1966). The criteria set forth by these authors for accepting rats into the diabetic group were used. Throughout the experiment these rats were fed a standard cubed diet and drinking water was supplied *ad libitum* in the form of a 2·5 per cent dextrose solution.

The experimental tumour system used in this investigation was the Walker 256 rat carcinosarcoma. It was prepared in the form of a sterile suspension of single tumour cells in Hank's balanced salt solution using a modification of the method described by Rodin, Turner and Couves (1963). The final concentration of this freshly prepared suspension was adjusted to contain 5000 viable tumour cells per 0·1 ml. of solution.

The rats were divided into 3 groups.

*Group* 1.—There were 10 rats in this group. They received no treatment other than injections of alloxan monohydrate intraperitoneally. They were retained for 16 days following the last intraperitoneal injection. At this stage they were rendered unconscious by ether anaesthetic and killed by exsanguination following cardiac puncture. The rats had been deprived of food and drinking water in the 12 hours immediately preceding death. The sugar concentration of the blood removed by cardiac puncture was determined by Nelson's modification of Somogy's method (Nelson, 1944).

*Group* 2.—A total of 41 rats were used. Twenty animals made up a control group and these animals were given intraperitoneal injections of sterile water in place of alloxan monohydrate. The control rats were maintained on a standard cubed diet and water was supplied *ad libitum*. The remaining 21 rats were treated with intraperitoneal alloxan monohydrate and formed the test group. Forty-eight hours after the last intraperitoneal injection all the rats had the left hind limb shaved and following cleaning of the overlying skin all the animals in the control group and the test group were given an intramuscular injection of 0·1 ml. of the freshly prepared tumour cell suspension into the posterior thigh muscle. Fourteen days after the intramuscular cell injections all the animals were rendered unconscious by ether anaesthetic and immediately killed by exsanguination following cardiac puncture. Food and drinking water had been withdrawn 12 hours before death. The hind limb tumours were exposed by removing the overlying skin and surrounding muscle and the maximum diameter of each limb tumour was estimated using a measuring calliper. The blood sugar level at the time of death was determined.

*Group* 3.—There were 41 rats in this group. As in the previous experiment a control group of 20 rats maintained on a standard cubed diet with drinking water supplied *ad libitum*, received injections of distilled water intraperitoneally. A test group of 21 rats was treated with intraperitoneal alloxan monohydrate.

In this experiment the freshly prepared tumour cells were " aged " under aseptic conditions for 12 hours at room temperature after the method described by Chan, Hadden, McDonald and Cole (1961). Their experimental results indicate that " aging " cancer cells for 6 to 12 hours will reduce the percentage of tumours produced following cell injection to a level somewhere between 60 and 30 per cent. This technique is of great value when it is not known whether a certain procedure or substance will increase or decrease the development of tumours after inoculation of cancer cells.

All the rats in the test and control groups were given a single intravenous injection of 0·1 ml. of the " aged " tumour cell suspension into the exposed saphenous vein found on the inner aspect of the hind limb 48 hours after the last intraperitoneal injection. The cells were injected under ether anaesthesia. The animals were retained for 14 days after the intravenous cell injections and were then killed by exsanguination following cardiac puncture under ether anaesthetic. As with the previous groups, food and drinking water was withdrawn 12 hours before death. They were subjected to careful post-mortem examinations to determine the presence of tumours. Macroscopic appearances were noted and selected tissues were excised for microscopic study. The blood sugar level at the time of death was determined.

### RESULTS

All the rats in the three groups remained in good condition throughout the duration of the experiment. It was noticeable, however, that the food and fluid intake of the alloxan diabetic rats was in excess of that of the non-diabetic animals.

The mean blood sugar value for the 10 rats in group 1 at the time of death was 230 mg. per cent with a standard deviation of 33 mg. per cent.

The experimental findings for group 2 are shown in Table I. The developing limb tumours were all palpable in the non-diabetic control rats 6 days after the

TABLE I.—*The Effect of Alloxan Induced Diabetes on Solid Tumour Growth Rate and the Effect of Developing Solid Limb Tumours on the Blood Sugar Level of Alloxan Diabetic Rats. Recordings made After* 14 *Days of Tumour Growth*

| | Number of rats | Mean maximum tumour diameter ± S.D. (mm.) | Mean blood sugar ± S.D. (mg.%) |
|---|---|---|---|
| Control Group | 20 | 36±8·7 | 96±15 |
| Alloxan diabetic group | 21 | 20±3·5 | 160±28 |

intramuscular tumour cell injections. In the diabetic group all the developing limb tumours were palpable 8 days after the cell injections. At the time of death the mean maximum tumour diameter for the non-diabetic animals was 36 mm. The mean maximum diameter for the alloxan diabetic group of animals was found to be significantly less at 20 mm. The mean blood sugar value for the non-diabetic control group of rats was 96 mg. per cent at the time of death. The normal range of blood sugar values for untreated rats in our laboratory when killed, following 12 hours deprivation of food and drinking water, is 75–100 mg. per cent. The mean blood sugar value for the alloxan diabetic, tumour bearing, rats at the time of death was 160 mg. per cent. While this blood sugar value is greater than that the mean blood sugar value for the non-diabetic tumour bearing animals, it is less than the mean blood sugar value determined for the alloxan diabetic, but non-tumour bearing, group of rats (group 1).

TABLE II.—*The Effect of Alloxan Induced Diabetes on the Development of Tumour Metastases from Circulating Cancer Cells and the Effect of the Tumour Metastases on the Blood Sugar Level of Alloxan Diabetic Rats. Recordings made* 14 *Days After the Intravenous Injection of Cancer Cells.*

| | | Metastases present | | Metastases absent | |
|---|---|---|---|---|---|
| | Number of rats | Number of rats | Mean blood sugar ± S.D. (mg.%) | Number of rats | Mean blood sugar ± S.D. (mg.%) |
| Control group | 20 | 11 | 87±12 | 9 | 76±11 |
| Alloxan diabetic group | 21 | 5 | 103±12 | 16 | 210±42 |

The experimental results for group 3 are shown in Table II. Pulmonary deposits of the Walker 256 tumour were found in 11 of the 20 rats in the non-diabetic control group at post-mortem examination. Five of the 21 rats in the diabetic group has tumour deposits within the lungs. These figures represent the findings on both macroscopic and microscopic examination of the lungs. In the diabetic group of animals the pulmonary tumours were smaller and more discrete than in the non-diabetic animals. In neither the control group nor the test group were tumour deposits found at any site other than the lungs. There was, therefore, a 55 per cent positive tumour take in the non-diabetic rats and a 24 per cent positive tumour take in the diabetic rats. The mean blood sugar value at the time of death in the 11 non-diabetic rats with pulmonary metastases was within normal limits at 87 mg. per cent. The mean blood sugar value for the 5 diabetic rats with pulmonary metastases was slightly elevated at 103 mg. per cent. The mean blood sugar value for the 9 rats in the control group that failed to develop tumour metastases from the circulating cancer cells was 76 mg.

per cent and is within the normal range. The mean blood sugar value for the 16 alloxan diabetic rats that failed to develop tumour metastases was 210 mg. per cent and this value is comparable to the mean blood sugar value found for the rats in group 1.

### DISCUSSION

From the results of this investigation, it is apparent that alloxan diabetic rats fare better than control rats in terms of rate of tumour growth and secondary tumour development. Not only do solid tumours in an intra-muscular situation grow more slowly in diabetic rats but diabetic rats also develop fewer pulmonary metastases than non-diabetic, control, animals following the intravenous injection of the same number of cancer cells.

This experimental study, however, throws no light on the mechanisms responsible for these observed results. The presence of diabetes in a tumour bearing animal may retard solid tumour growth indirectly by depriving the developing cancer of vital metabolites or the diabetic process may act directly on the cancer cells, involving them in the metabolic abnormalities occurring elsewhere within the host. The associated hyperglycaemia of diabetes may be of some protective value in preventing the development of tumour metastases from circulating cancer cells. It has been previously shown that if rats are made hyperglycaemic by the intraperitoneal injection of glucose before the intravenous injection of cancer cells, then these animals will develop significantly fewer tumour metastases than rats that have normal blood sugars at the time the tumour cells are introduced into the circulation (Garvie, 1967).

Should the results of this experimental investigation be applicable to the human situation, then patients with diabetes mellitus who develop malignant disease should fare better in terms of survival than non-diabetic patients who develop a similar cancer. However, certain factors require consideration. Patients with established diabetes who develop malignant disease will be receiving treatment for their diabetes and, although it is not known how such treatment will influence the malignant process, it is to be anticipated that this therapy will make the diabetic patient react to his malignant disease in a manner similar to the non-diabetic patient. On the other hand, in both diabetes mellitus and cancer there may be a considerable time interval between the inception of the disease and its clinical recognition. From estimations based on epidemiological data Anderson (1966) has shown that for persons in their forties and fifties the mean length of time between the appearance of impaired carbohydrate tolerance and the time of diagnosis of diabetes mellitus is at least 10–12 years. The period of undetected growth for malignant tumours similarly spans many years (Schwartz, 1961). It is apparent, therefore, that these two diseases can co-exist undiagnosed within the same host for some considerable time and under these circumstances the diabetes may have a modifying influence on the malignant process.

The tumour bearing rats also fare better with respect to their diabetes than the non-tumour bearing, but diabetic, control animals. The results of this investigation show that the presence of a developing cancer within the diabetic host animal has a modifying effect on the hyperglycaemia of the diabetic process. Ingle (1960) has found that in rats the glycosuria of steroid induced diabetes is suppressed if the animals are made hosts to the Walker 256 tumour. He concluded that the improvement in the diabetic state could be attributed either to a non-specific toxic effect of the tumour on general metabolic activity or that the tumour might produce a more direct metabolic effect such as cortisone inactivation. However, in spite of further research into this problem the reason for the improvement in the diabetic state remains undetermined (Ingle, 1965).

The results of this investigation have shown that in rats the two disorders, alloxan diabetes and cancer have a modifying influence on each other. The human situation is currently being investigated to see if a similar relationship between diabetes mellitus and cancer exists.

SUMMARY

The influence of alloxan induced diabetes on tumour growth rate and secondary tumour development has been estimated, using rats as the experimental animal. It has been shown that not only does alloxan induced diabetes retard the rate of tumour growth but also that it inhibits the development of metastases from circulating cancer cells. The possible clinical significance of these findings are discussed.

It was also found that the presence of a developing cancer in alloxan diabetic rats had a modifying effect on the associated hyperglycaemia. The reason for this improvement in the diabetic state is not known.

### REFERENCES

ANDERSON, T. W.—(1966) *Diabetes*, **15**, 160.

CARIDIS, D. T., BLAIR, D. W., KILPATRICK, S. J. AND CARR, A. J.—(1966) *Scott. med. J.*, **11**, 247.

CHAN, P. Y. H., HADDEN, D. R., McDONALD, G. O. AND COLE, W. H.—(1961) *Cancer, N.Y.*, **14**, 1057.

GARVIE, W. H. H.—(1967) *Br. J. Surg.*, **54**, 229.

INGLE, D. J.—(1960) *Endicrinology*, **66**, 289.—(1965) *Diabetes*, **14**, 93.

NELSON, N. A.—(1944) *J. biol. chem.*, **153**, 375.

RODIN, A. E., TURNER, F. W. AND COUVES, C. M.—(1963) *Can. J. Surg.*, **6**, 489.

SCHWARTZ, M.—(1961) *Cancer, N.Y.*, **14**, 1272.